# Exposure to the Harmful Algal Bloom (HAB) Toxin Microcystin-LR (MC-LR) Prolongs and Increases Severity of Dextran Sulfate Sodium (DSS)-Induced Colitis

**DOI:** 10.3390/toxins11060371

**Published:** 2019-06-25

**Authors:** Robin C. Su, Thomas M. Blomquist, Andrew L. Kleinhenz, Fatimah K. Khalaf, Prabhatchandra Dube, Apurva Lad, Joshua D. Breidenbach, Chrysan J. Mohammed, Shungang Zhang, Caitlin E. Baum, Deepak Malhotra, David J. Kennedy, Steven T. Haller

**Affiliations:** 1 Department of Medicine, The University of Toledo College of Medicine and Life Sciences, Toledo, OH 43614, USA; Robin.Su@rockets.utoledo.edu (R.C.S.); Andrew.Kleinhenz@utoledo.edu (A.L.K.); Kareem.Khalaf@rockets.utoledo.edu (F.K.K.); Prabhatchandra.Dube@utoledo.edu (P.D.); Chrysan.Mohammed@rockets.utoledo.edu (C.M.); Shungang.Zhang@rockets.utoledo.edu (S.Z.); Deepak.Malhotra@utoledo.edu (D.M.); 2 Department of Pathology, The University of Toledo College of Medicine and Life Sciences, Toledo, OH 43614, USA; Thomas.Blomquist@utoledo.edu (T.M.B.); Caitlin.Baum@utoledo.edu (C.E.B.); 3 Department of Medical Microbiology and Immunology, The University of Toledo College of Medicine and Life Sciences, Toledo, OH 43614, USA; Apurva.Lad@rockets.utoledo.edu (A.L.); Joshua.Breidenbach@rockets.utoledo.edu (J.D.B.)

**Keywords:** inflammatory bowel disease, dextran sulfate sodium, colitis, microcystin, colon

## Abstract

Inflammatory Bowel Disease (IBD) represents a collection of gastrointestinal disorders resulting from genetic and environmental factors. Microcystin-leucine arginine (MC-LR) is a toxin produced by cyanobacteria during algal blooms and demonstrates bioaccumulation in the intestinal tract following ingestion. Little is known about the impact of MC-LR ingestion in individuals with IBD. In this study, we sought to investigate MC-LR’s effects in a dextran sulfate sodium (DSS)-induced colitis model. Mice were separated into four groups: (a) water only (control), (b) DSS followed by water (DSS), (c) water followed by MC-LR (MC-LR), and (d) DSS followed by MC-LR (DSS + MC-LR). DSS resulted in weight loss, splenomegaly, and severe colitis marked by transmural acute inflammation, ulceration, shortened colon length, and bloody stools. DSS + MC-LR mice experienced prolonged weight loss and bloody stools, increased ulceration of colonic mucosa, and shorter colon length as compared with DSS mice. DSS + MC-LR also resulted in greater increases in pro-inflammatory transcripts within colonic tissue (TNF-α, IL-1β, CD40, MCP-1) and the pro-fibrotic marker, PAI-1, as compared to DSS-only ingestion. These findings demonstrate that MC-LR exposure not only prolongs, but also worsens the severity of pre-existing colitis, strengthening evidence of MC-LR as an under-recognized environmental toxin in vulnerable populations, such as those with IBD.

## 1. Introduction

Inflammatory bowel disease (IBD) is a collection of disorders characterized by both acute and chronic inflammation of the gastrointestinal (GI) tract [1]. IBD has become a global health burden, with an estimated 1 million individuals in the USA and 2.5 million individuals in Europe being affected by IBD [2]. Total indirect and direct costs of IBD in the US were estimated to have been between $14.6 and $31.6 billion in 2014 [3] and a recent study found total costs of IBD patients in 2015 to be three times higher than non IBD patients [4]. In addition, IBD is rapidly growing in prevalence within newly industrialized countries around the world [2]. 

Two of the most common forms of IBD are Crohn’s disease (CD) and ulcerative colitis (UC), which share certain characteristics but also exhibit key differences. CD can affect any region of the GI tract and is characterized by discontinuous “skip lesions” while UC most commonly manifests within the distal GI tract, starting in the rectum and progressing proximally in a continuous manner along the distal colon [5]. Hallmarks of CD are transmural acute and chronic inflammation, non-caseating granulomas, strictures, and fistulas, while UC is characterized by mucosal and submucosal acute inflammation, crypt abscesses, ulcerations, depletion of goblet cells and mucin, bloody diarrhea, and weight loss [1]. These two conditions are frequently accompanied by other comorbidities and complications, making overall clinical presentations complex, difficult to manage, financially burdensome, and symptomatically debilitating for affected individuals. 

IBD is a complex disease with a multifactorial etiology. There are genetic components that predispose individuals to develop IBD, as well as a dysregulation within the host immune system and GI microbial environment [6]. However, there is also an important role that the environment plays in IBD’s pathogenesis and disease severity. This is highlighted by its dominant prevalence in industrialized countries and its growing prevalence in newly developed countries [6]. Many of these environmental factors have been explored, including environmental sanitation and hygiene; behavioral factors, such as smoking, diet, stress management, and breastfeeding; and use of medications, such as antibiotics, non-steroidal anti-inflammatory drugs, and oral contraceptives [6]. Additional environmental factors include microorganism infections, such as those caused by *Helicobacter pylori*, *Mycobacterium avium*, *Escherichia coli*, *Yersinia enterocolitica*, *Listeria monocytogenes*, and *Candida albicans* [6]. The identification of these different triggers of IBD disease progression have allowed for the establishment of appropriate preventative and therapeutic measures, however, there is still an urgent need to continue investigating other potential offenders.

One growing global environmental concern that has not been studied for its effects in IBD populations is microcystin. Microcystins (MCs) are a collection of potent toxins produced by cyanobacteria, also known as blue-green algae [7]. Of these toxins, microcystin-LR (MC-LR) is one of the most commonly produced forms and is also one of the most toxic variants [8]. Harmful algal blooms (HABs) contaminate freshwater environments and have affected every region of the USA. Globally, more than 40% of lakes and reservoirs in Europe, Asia, and America have favorable conditions for HABs, with 25–75% of blooms being considered toxic [9]. In addition, these HABs are increasing exponentially in frequency and severity worldwide [10]. The acute and chronic effects of microcystin exposure in humans have been recently reviewed [11]. Notable events of MC-LR exposure and toxicity in humans have been documented around the world. One of the most notable events occurred in 1996, where 116 of 130 patients at a dialysis center experienced acute liver failure and death within one week of exposure to water sources contaminated with microcystin [12,13]. Previous studies have identified the gastrointestinal tract to be a potentially important target of MC-LR toxicity and have even shown the intestines to be the site of greatest MC-LR bioaccumulation [10,14,15,16]. While MC-LR has been shown to cause severe liver damage [15], found to be a potential human carcinogen [8], and documented to be fatal in humans in some complicated cases [12], there is a critical need to investigate MC-LR’s effects within the intestines, especially in more vulnerable settings, such as IBD. 

In this current study, we aimed to address these pressing gaps in knowledge by utilizing the well-established dextran sulfate sodium (DSS) model of colitis in C57BL/6 mice [17]. This model has been developed to mimic characteristics of both CD and UC, and has been extensively validated by the use of several therapeutic agents used to treat human IBD [18]. The DSS model induces acute colitis and has been shown to sustain chronic levels of inflammation [19]. Because of MC-LR’s known bioaccumulation in the intestines, we hypothesized that MC-LR would prolong and/or worsen the severity of DSS-induced colitis.

## 2. Results

### 2.1. Body Weight and Survival

**Figure 1:**
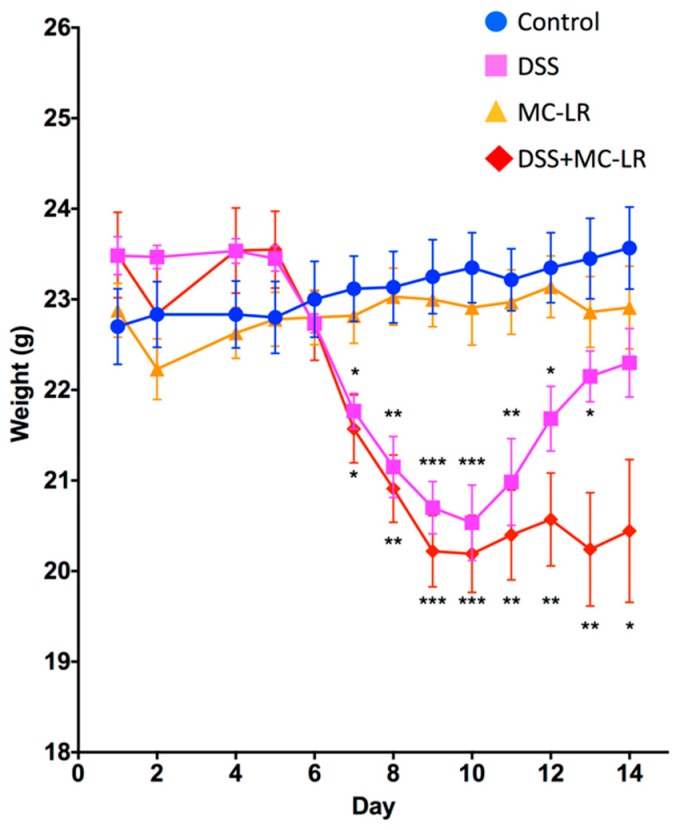
Mouse body weights taken daily throughout the 14-day study. Data presented indicate the mean ± SEM (n = 6–10 mice per group). * *p* < 0.05, ** *p* < 0.01, and *** *p* < 0.001 vs. the control group.

Mice in the MC-LR group showed no significant differences in weight throughout the 14-day study as compared with control mice. Mice in the DSS group and in the DSS + MC-LR group showed decreases in body weight starting at day 6 (Figure 1). Starting at day 7, the body weights of DSS and DSS + MC-LR mice were significantly lower than the control and MC-LR mice (*p* < 0.05). No differences were observed between DSS and DSS + MC-LR mice. Body weights of the DSS and DSS + MC-LR mice continued to decrease until day 10 (*p* < 0.001). DSS mice progressively regained their weight starting at day 11. At day 13, DSS mice had significantly greater body weights than DSS + MC-LR mice. DSS mice continued to regain their weight until day 14, at which point they showed no significant differences in body weight versus the control and MC-LR mice, but were significantly greater in body weight versus DSS + MC-LR mice. While DSS mice regained their weight, mice in the DSS + MC-LR group continued to show significantly lower body weights until day 14 as compared with the control and MC-LR mice (*p* < 0.05). One mouse in the DSS + MC-LR group was found dead on day 14. Analysis on this mouse was not possible as organs were not attainable through standard procedures.

### 2.2. Stool Grading

**Figure 2:**
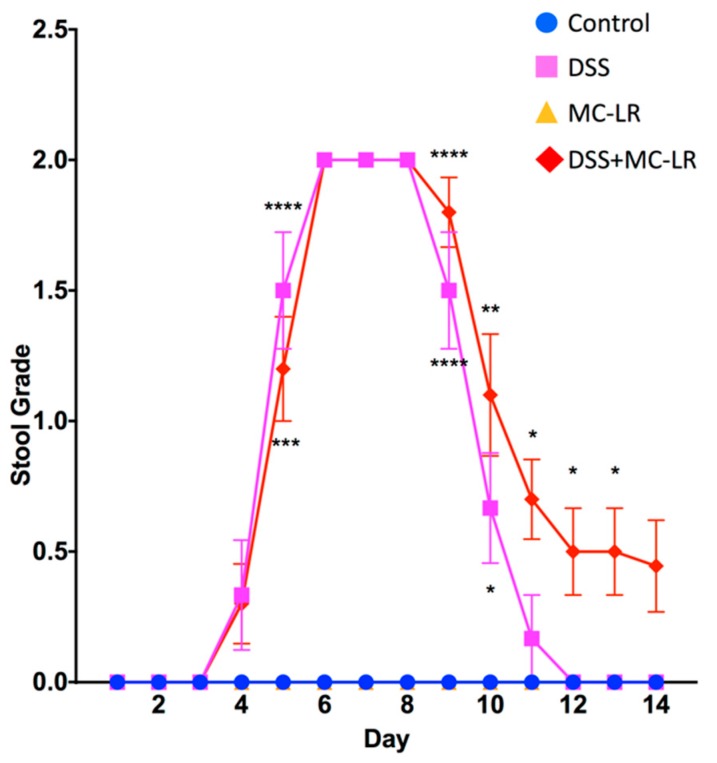
Daily stool grading throughout the 14-day study. 0 = no occult or gross blood, 1 = occult blood present, and 2 = gross blood present. Data presented indicate the mean ± SEM (n = 6–10 mice per group). * *p* < 0.05, ** *p* < 0.01, *** *p* < 0.001, and **** *p* < 0.0001 vs. control group.

Mice in the control and MC-LR groups showed no occult or gross blood in their stool throughout the 14-day study (Figure 2). Mice in the DSS group and in the DSS + MC-LR group began showing signs of occult blood in their stool on day 4 and gross blood starting on day 5. No significant differences in the occurrence of occult blood was observed between DSS and DSS + MC-LR groups. All mice in both DSS and DSS + MC-LR groups exhibited gross blood in their stool from days –8. Mice in DSS and DSS + MC-LR groups showed a decrease in gross blood and in increase in occult blood in their stool starting on day 9. DSS mice showed complete resolution of gross and occult blood by day 10. By day 11, there was a significantly lower occurrence of occult blood in the DSS group as compared with the DSS + MC-LR group (Figure 2). DSS + MC-LR mice showed no resolution of blood in their stool, with persistent occult blood still being detectable at day 14.

### 2.3. Colon Length and Spleen Weight

**Figure 3:**
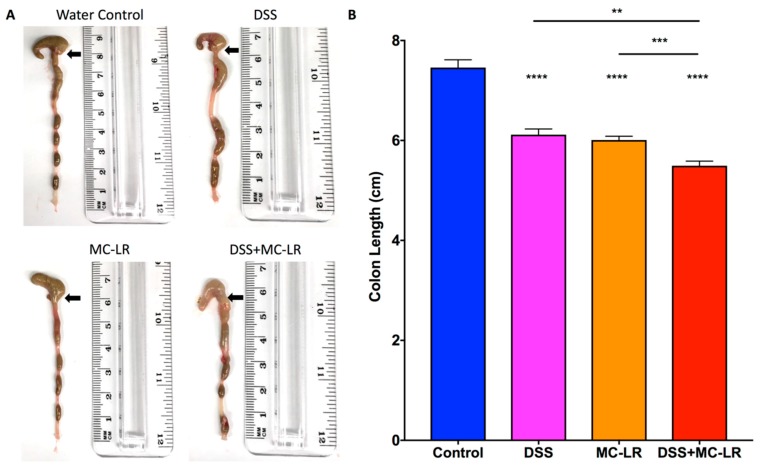
Effect of DSS and MC-LR on colon length. (**A**) Representative gross images and measurements of mouse colons with cecums still attached. Black arrows indicate the colon-cecum junction as a landmark for colon length measurement. (**B**) Diagram of colon lengths. Data presented indicate the mean ± SEM (n = 6–10 mice per group). ** *p* < 0.01, *** *p* < 0.001, and **** *p* < 0.0001.

Colons were measured from the distal end to the colon-cecum junction as exemplified by the representative images in Figure 3A. Colons of mice in the DSS, MC-LR, and DSS + MC-LR groups showed significant decreases in length as compared with the control colons (*p* < 0.0001) (Figure 3B). It was also noted that the colon lengths of the DSS + MC-LR mice were significantly shorter than those in the DSS (*p* < 0.01) and MC-LR groups (*p* < 0.001). 

**Figure 4:**
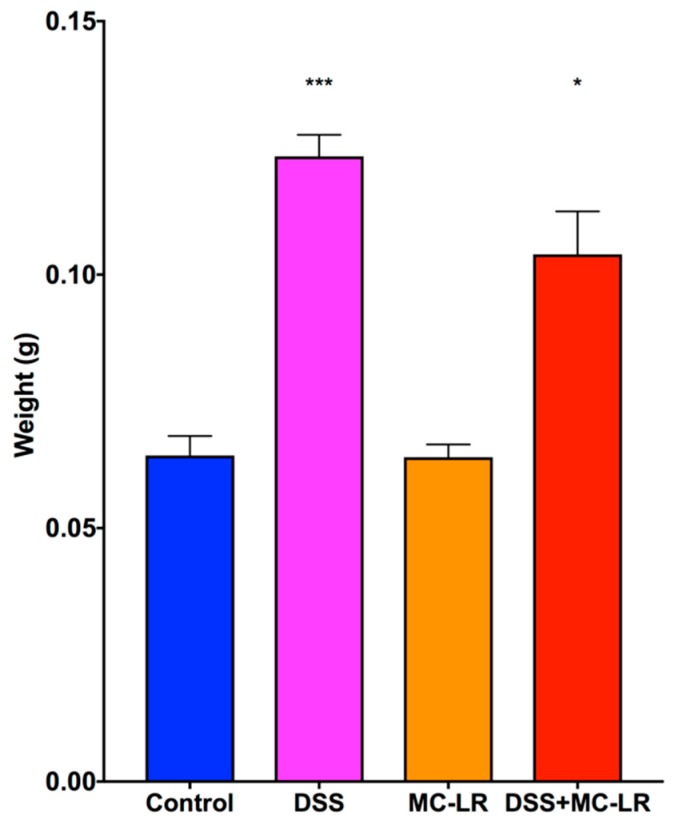
Spleen weights measured at the time of organ harvesting. Data presented indicate the mean ± SEM (n = 6–10 mice per group). * *p* < 0.05, and *** *p* < 0.001 vs. control group.

Spleen weights were significantly greater in the DSS group (*p* < 0.001) and the DSS + MC-LR group (*p* < 0.05) as compared with the control group (Figure 4). No differences were observed in spleen weight between the DSS and DSS + MC-LR groups. Spleen weights in the MC-LR group were not increased as compared with the control group.

### 2.4. Histopathology

**Figure 5:**
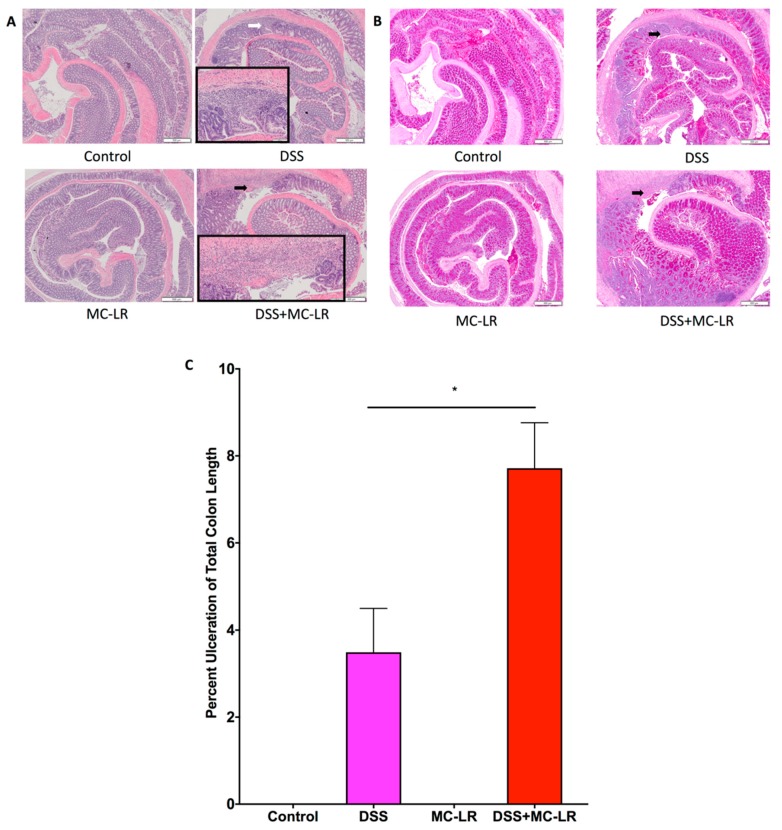
Effect of DSS and MC-LR on histopathological changes. (**A**) Representative images of H&E stained colon sections. Colon tissue from control and MC-LR mice did not show any specific pathologic changes. DSS exposure led to disruption of the epithelium, segmental regions of marked acute inflammatory cell infiltration, ulceration and branching and budding of glands (early chronic changes). A white arrow demarcates a representative area of tissue ulceration that has begun early re-epithelialization (magnified in the black framed area) and shows obliteration of crypts and infiltration by inflammatory cells. DSS + MC-LR combined exposure demonstrates increased severity of the same histopathological changes seen in the DSS group. A black arrow demarcates a representative area of tissue ulceration (magnified in the black framed area), which is significantly larger in length than that found in the DSS group. Scale Bar: 100 μm. (**B**) Representative PAS stained colon sections (same samples as the representative H and E sections). Colon tissue from control and MC-LR mice show continuous staining of goblet cells and mucin throughout the length of the colon. DSS exposure led to the loss of goblet cells and mucin, especially flanking the areas of ulceration (black arrow). The decrease in goblet cells and mucin depletion is more exaggerated in the DSS + MC-LR group (black arrow). Scale Bar: 100 μm (**C**) Quantification of tissue ulceration. Total ulcer length throughout the colon was normalized to total colon length. Data presented indicate the mean ± SEM (n = 6–10 mice per group). * *p* < 0.05 vs. the DSS group.

Histopathological analysis of hematoxylin and eosin (H & E) stained colon sections revealed that DSS exposure led to segmental regions of ulceration, crypt abscesses, marked acute inflammatory cell infiltration, and early architectural distortion with gland branching and budding (i.e., early chronic changes), as compared with the normal colonic tissue of the control group (Figure 5A). Analysis of Periodic acid–Schiff (PAS) stained colon sections highlighted loss of goblet cells with mucin depletion, especially within and flanking the ulcer beds (Figure 5B).

H and E analysis of the colons in the DSS + MC-LR group revealed greater severity of the same histopathological findings observed in the DSS group. Measurement of the length of ulcerated mucosa to total colon length demonstrates a greater percentage of ulceration within the DSS + MC-LR group as compared to the DSS group (Figure 5C). The MC-LR and control groups did not show evidence of pathological changes.

### 2.5. Gene Expression in the Colon

**Figure 6:**
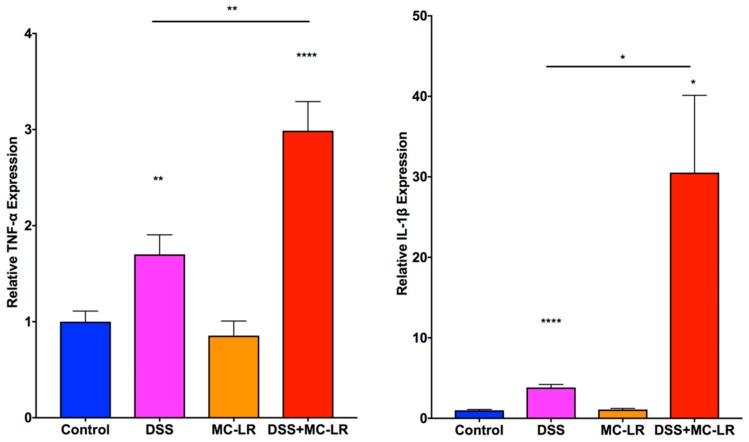
RT-qPCR analysis of proinflammatory cytokines TNF-α and IL-1β mRNA. Data presented indicate the mean ± SEM (n = 6–10 mice per group). * *p* < 0.05, ** *p* < 0.01, and **** *p* < 0.0001 vs. the control group.

As seen in Figure 6, qPCR analysis demonstrated that the mRNA levels of the pro-inflammatory cytokines TNF-α and IL-1β were significantly upregulated in the DSS group as compared with the control group (*p* < 0.01 and *p* < 0.0001, respectively). The upregulation of TNF-α and IL-1β was further increased in the DSS + MC-LR group (*p* < 0.0001 and *p* < 0.05, respectively) and were significantly higher than the levels observed in the DSS group (*p* < 0.01 and *p* < 0.05, respectively).

**Figure 7:**
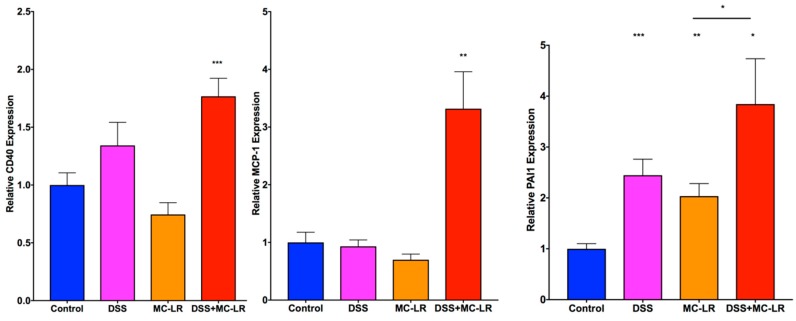
RT-qPCR analysis of CD40 and its downstream products MCP-1 and PAI-1. Data presented indicate the mean ± SEM (n = 6–10 mice per group). * *p* < 0.05, ** *p* < 0.01, and *** *p* < 0.001 vs. the control group.

CD40 mRNA levels (Figure 7) were increased in the DSS group and significantly elevated in the DSS + MC-LR group compared to control (*p* < 0.001). CD40 expression was not elevated in the MC-LR group.

Expression of MCP-1 (Figure 7) was significantly upregulated in the DSS + MC-LR group (*p* < 0.01). PAI-1 was upregulated within the DSS (*p* < 0.001), MC-LR (*p* < 0.01), and DSS + MC-LR (*p* < 0.05) groups as compared with the control group. PAI-1 levels were significantly higher in the DSS + MC-LR group as compared with the MC-LR group (*p* < 0.05).

## 3. Discussion

This study is the first to describe MC-LR’s effects within the gastrointestinal tract of mice with pre-existing colitis. Here, we demonstrate that MC-LR can prolong and potentiate the severity of colitis within a DSS colitis mouse model. By utilizing a well-established colitis protocol, we found DSS to induce weight loss, splenomegaly, and severe colitis marked by transmural acute inflammation, ulceration, shortened colon length, and bloody stools. These gross effects were accompanied by the upregulation of key pro-inflammatory transcripts within colonic tissue, including TNF-α and IL-1β. While MC-LR alone only resulted in modest colonic shortening and increases in PAI-1 expression, MC-LR in the setting of DSS-induced colitis resulted in the prolongation and exacerbation of disease state as compared with DSS alone. Within the DSS + MC-LR group, we observed prolonged weight loss and bloody stools, increased ulceration of colonic mucosa, shorter colon length, and a greater increase in the pro-inflammatory transcripts of TNF-α and IL-1β as compared with DSS alone. It is also important to note that the heightened disease state in the DSS + MC-LR group resulted in the death of one of the mice before the conclusion of the study, a finding not found in any of the other groups or reported in any other previous study.

It has been well established that MC-LR enters cells through organic anion transporting polypeptides (OATP) [20]. Once in cells, MC-LR inhibits serine/threonine protein phosphatases (PPs), especially PP1 and PP2A [20]. Such inhibition of PPs lead to hyperphosphorylation of various enzymes and cytoskeletal elements, leading to a disruption of cellular processes [20]. In Sertoli cells, it has been found that one of the consequences of MC-LR + PP complex formation is the inhibition of miR-98-5p and miR-758, leading to the enhanced expression of MAPK11 [21]. Enhanced expression and activation of MAPK11 leads to the phosphorylation of transcription factor ATF-2, which binds to the promotor of TNF-α and leads to TNF-α expression [21].

Another potential mechanism by which MC-LR enhances inflammatory cytokine production has recently been investigated by Adegoke et al. [22]. Again in Sertoli cells, it was found that MC-LR induces the upregulation and activation of toll-like receptor 4 (TLR4) and its downstream effector, nuclear factor-kappaB (NF-kB), in a dose-dependent manner [22]. It has been proposed that the activation of NF-kB by TLR4 is mediated by either myeloid differentiation primary response 88 (MyD88) or TIR-domain-containing adapter-inducing interferon-β (TRIF) [22]. NF-kB activation subsequently leads to the upregulation of pro-inflammatory cytokines, including TNF-α and IL-1β [22]. A follow up study by Adegoke et al. utilized TLR4-IN-C34 (C34) to inhibit TLR4 in order to demonstrate an attenuation of MC-LR toxicity by the TLR4/NF-kB pathway [23]. Inhibition with C34 was found to attenuate damage caused by MC-LR and attenuate the production of inflammatory cytokines, including TNF-α and IL-1β [23].

**Figure 8:**
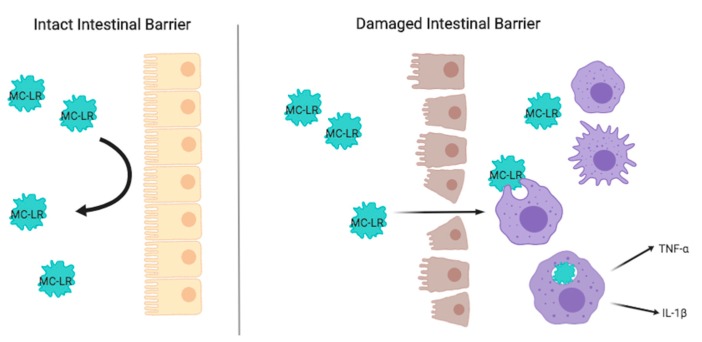
Schematic diagram displaying a potential mechanism by which MC-LR entry is facilitated by intestinal epithelial damage. An intact intestinal barrier helps to prevent the tissue uptake of foreign matter, including MC-LR. A damaged intestinal barrier may facilitate the tissue uptake of MC-LR. MC-LR, like other foreign material is scavenged by macrophages, which release inflammatory cytokines, such as TNF-α and IL-1β, and further recruits additional innate immune cells driving inflammation.

In addition, MC-LR activates the innate immune system by the recruitment of lymphocytes, neutrophils, and macrophages into affected tissues [24,25]. Such activation leads to downstream cytokine production, including TNF-α and IL-1β by macrophages. The upregulation of TNF-α and IL-1β due to MC-LR exposure has been previously confirmed [26]. Given that disruption of the intestinal epithelial barrier serves as a major driver of IBD pathogenesis [27], our results may highlight the importance of intestinal barrier integrity in MC-LR toxicity. Pre-existing colitis, coupled with barrier dysfunction, could lead to greater tissue uptake of MC-LR, greater recruitment of innate immune cells, and greater production of inflammatory cytokines, a phenomenon that is not activated in the setting of intact intestinal barriers in healthy wild type mice. This concept is illustrated in Figure 8.

As CD40 signaling has been recently implicated in active regions of IBD in human patients, we evaluated CD40 as a potential mechanism for this disease prolongation and exacerbation [28]. CD40 is part of the TNF superfamily, one of three families that can be exploited in T cell co-stimulation [29]. CD40 has been evaluated in the setting of IBD (both UC and CD) and has been found to be overexpressed in IBD. Interestingly, previous studies in IBD patients have shown that CD40 overexpression is directly proportional to the extent of disease severity and is only found in actively inflamed regions of intestinal tissue [30]. In addition to their overexpression on T cells, CD40 has also been shown to be expressed on intestinal fibroblasts and on intestinal epithelial cells in actively inflamed colonic tissue of IBD patients, which was further confirmed in a cell model [28,31]. In the present study, we observed a significant upregulation of CD40 in the DSS + MC-LR colons, signifying its potential role in disease exacerbation. Of note, we also observed the upregulation of MCP-1 and PAI-1, two downstream products of CD40 activation. These help to confirm the initiation of this pathway as a result of MC-LR exposure in the setting of DSS-induced colitis.

In this study, it is important to also note the relevance of the dosage of MC-LR used. The World Health Organization (WHO) has set a limit on permissible levels of microcystin in finished drinking water at one part per billion (ppb), which is commonly understood as 1 μg/kg [32]. The WHO has also established that when conducting research in an animal model, an uncertainty factor of 1000 can be applied in order to account for intra- and interspecies variation, allowing for 1 ppb to be extrapolated to 1000 μg/kg in an animal model [33], which is the concentration we utilized in the present study. In addition, this dose of 1000 μg/kg has previously been studied by Fawell et al. [34]. While our current study exposed mice to 1000 μg MC-LR/ kg body weight daily for one week, Fawell et al. exposed mice to 40, 200, and 1000 μg MC-LR/ kg body weight daily for 13 weeks, a prolonged, chronic exposure timeframe. While no significant increases in pathology was noted in the 40 μg/kg male mouse exposure group as compared with controls after 13 weeks, increased trends in liver pathology (including chronic inflammation, hepatocyte degeneration, and hemosiderin deposition), and blood chemistry (including alanine aminotransferase and aspartate aminotransferase) were noted in the 200 and 1000 μg/kg male mouse exposure groups at the conclusion of 13 weeks of daily MC-LR exposure [34]. While these hepatic findings may be significant with chronic exposure (13 weeks) in healthy mouse models, we aimed to investigate the gastrointestinal effects of acute exposure (one week) to MC-LR. Interestingly, while we saw limited effects in WT mice, MC-LR had significant effects in mice with pre-existing colitis. Our findings suggest that more stringent regulations for MC-LR in finished drinking water should be considered in order to protect populations that may be more susceptible to MC-LR toxicity, such as those with pre-existing gastrointestinal diseases.

In summary, while MC-LR exposure alone was not found to induce significant inflammation, histopathology, changes in stool, or changes in body or spleen weight, it was found to have a profound effect in the setting of pre-existing colitis by prolonging and exacerbating disease conditions. The use of relevant MC-LR exposure levels further highlights the clinical relevance and urgent need for stricter guidelines in order to protect vulnerable populations. Future studies will help to further elucidate the role of CD40 in DSS-induced colitis, its role in disease exacerbation in the presence of MC-LR exposure, and its potential as a therapeutic target for disease prevention and reversal.

## 4. Materials and Methods

### 4.1. Mice

All animal experimentation was conducted in accordance with the National Institutes of Health (NIH) Guide for the Care and Use of Laboratory Animals under protocols approved by The University of Toledo Institutional Animal Care and Use Committee (IACUC protocol #108663, approval date 9 February 2016). All mice were housed in a specific pathogen free facility, maintained at standard conditions of 23 ± 1 °C under a 12-h light cycle and were allowed to eat a normal chow diet ad libitum. Male C57BL/6 mice were purchased from The Jackson Laboratory at five weeks of age. The mice were immediately assigned randomly to one of four groups: (a) water only (control), (b) DSS followed by water (DSS), (c) water followed by MC-LR (MC-LR), and (d) DSS followed by MC-LR (DSS + MC-LR). The control water group consisted of 6 mice, the DSS group consisted of six mice, the MC-LR group consisted of 10 mice, and the DSS + MC-LR group consisted of 10 mice. All 32 mice were allowed to acclimatize to their new environment until eight weeks of age.

### 4.2. Colitis Induction and MC-LR Exposure Protocol

**Figure 9:**
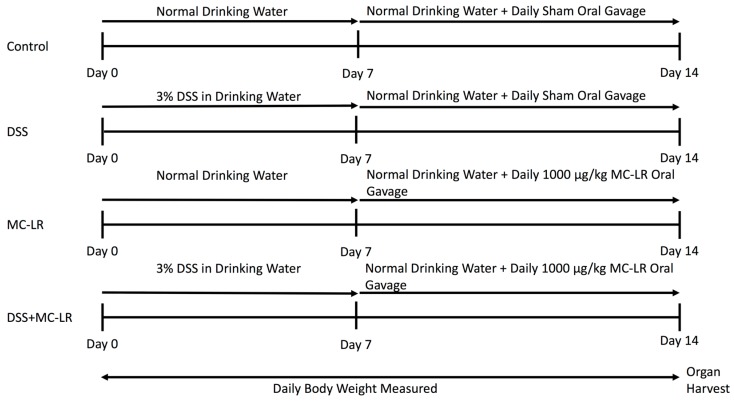
Experimental design evaluating the effects of DSS exposure, MC-LR exposure, and combined DSS + MC-LR exposure within colons of C57BL/6 mice. The study was conducted on eight-week old mice. DSS mice were given 3% DSS in drinking water for seven days followed by seven days of normal drinking water and daily sham oral gavage. MC-LR mice were given normal water for seven days followed by seven days of continued normal water and daily 1000 μg/kg MC-LR oral gavage. DSS + MC-LR mice were given 3% DSS in drinking water for seven days followed by seven days of normal drinking water and daily 1000 μg/kg MC-LR oral gavage. Body weights were measured daily and organ harvesting was conducted immediately after euthanasia.

At eight weeks of age, mice were initiated into the study as outlined in Figure 9. The control mice were allowed to drink water ad libitum for 14 days. Between days 8–14, control mice were orally gavaged daily with water as a sham procedure. To induce colitis, mice in the DSS group were given 3% DSS (MP Biomedical, Solon, OH, USA, Item No. 0216011080) in drinking water ad libitum for seven days according to established protocols [17]. Following this, the mice were allowed to drink untreated water ad libitum for 7 days, while also being given water by daily sham oral gavage. The MC-LR group of mice were allowed to drink water ad libitum for 14 days. During days 8–14, these mice were orally gavaged 1000 μg/kg MC-LR (Cayman Chemical, Ann Arbor, MI, USA, item no. 10007188) daily. The DSS + MC-LR group of mice were allowed to drink 3% DSS water ad libitum for seven days and then were allowed to drink untreated water ad libitum for seven days. During days 8–14, these mice were orally gavaged 1000 μg/kg MC-LR daily. Weight of each mouse was taken daily. Stool was evaluated daily for the presence of occult and gross blood. Occult blood was measured using the Beckman Coulter Hemoccult Single Slides kit (Med Plus Physician Supplies, Edison, New Jersey, USA, Catalog #BC-60151). Stool was graded by the following: 0 = no occult or gross blood, 1 = occult blood present, and 2 = gross blood present. All mice were euthanized on day 15 and organs were harvested and weighed immediately following euthanasia. Colons (with cecum still intact) were measured adjacent to a standard ruler and photographs were taken. Following removal of the cecum and thorough washing of the colon with PBS, sections of distal and proximal colon were taken from each sample, flash frozen in liquid nitrogen, and subsequently stored at −80 °C for future qPCR analysis.

### 4.3. Histology

Remaining colonic tissue from these mice were cut longitudinally, wrapped around a rigid holder, placed in cassettes, and fixed in 10% neutral buffered formalin for 24 h. The cassettes were then transferred to 70% ethanol. This formalin fixed tissue was then processed and embedded in paraffin (FFPE). Five (5) micron tissue sections were placed on glass slides and stained with hematoxylin and eosin (H & E) and Periodic acid–Schiff (PAS). Images of histology slides were taken using an Olympus CKX53 microscope and Olympus CellSens software (Standard 1.15) (Center Valley, PA, USA).

Severity of colon ulceration was further quantified using the Olympus CellSens software by measuring total length of ulcerated colon and normalizing to the total length of colon to give percent of colonic mucosa with ulceration.

### 4.4. RNA Extraction and RT-qPCR Method

RNA extraction, cDNA preparation, and RT-qPCR were all performed utilizing the QIAGEN (Germantown, MD, USA) automated liquid handling workflow system (QIAcube HT and QIAgility). RNA from distal colonic tissue was isolated utilizing the QIAzol/chloroform extraction methodology. RNA was purified using the lithium chloride method as previously published [35]. Approximately 500 ng of extracted RNA was used to synthesize cDNA (QIAGEN’s RT2 First Strand Kit). RT-qPCR was performed utilizing QIAGEN’s Rotor-Gene Q thermo-cycler. Calculation of gene expression was conducted by comparing the relative change in cycle threshold value (ΔCt). Fold change in expression was calculated using the 2-ΔΔCt equation as previously described [36]. The following Taqman primers were used and obtained from Thermo Fisher Scientific: TNF-α (Mm00443258_m1), IL-1β (Mm00434228_m1), PAI-1 (Mm00435858_m1), MCP-1 (Mm00441242_m1), and CD40 (Mm00441891_m1). 18s rRNA from Thermo Fisher Scientific was used as a housekeeping gene for normalization of transcript expression (catalog no. 4319413E).

### 4.5. Statistical Analysis

All data is presented as mean ± SEM. Statistical analysis was conducted with GraphPad Prism 7.0d software (San Diego, CA, USA) using the unpaired two-tailed Student’s t-test. Significance was determined if *p* values were < 0.05.
